# Systematic reinstatement of highly sacred *Ficuskrishnae* based on differences in morphology and DNA barcoding from *Ficusbenghalensis* (Moraceae)

**DOI:** 10.3897/phytokeys.186.74086

**Published:** 2021-12-09

**Authors:** Karthikeyan Mahima, Senthilkumar Umapathy, Jana Venkata Sudhakar, Ramalingam Sathishkumar

**Affiliations:** 1 Plant Genetic Engineering Laboratory, Department of Biotechnology, Bharathiar University, Coimbatore–641 046, Tamil Nadu, India Bharathiar University Coimbatore India; 2 Department of Plant Biology and Plant Biotechnology, Madras Christian College, Chennai- 600 059, Tamil Nadu, India Madras Christian College Chennai India; 3 Department of Botany, S.R.K Govt. Arts College, Pondicherry University–533 464, Tamil Nadu, India. Pondicherry University Pondicherry India

**Keywords:** Bayesian analysis, conspecifics, *Ficus*, ITS2 region, species delimitation

## Abstract

*Ficuskrishnae* is considered as native to India and is well-known for the peculiarity in nature of its cup-shaped leaves where both the vernacular name (Krishna Fig) and specific epithet were derived. The taxonomic status of *Ficuskrishnae* is still unclear and currently treated as a subspecies or variety under *Ficusbenghalensis*. In the present study, morphological characters and molecular analysis were employed to address their species delimitation. The spacer markers ITS2 and *trnH-psbA* were used for constructing phylogenetic trees along with morphometric analysis. *Ficuskrishnae* distinctly differs from *Ficusbenghalensis* by having cup-forming leaves and the nature of the aerial roots, stipules, petioles, ostiolar bracts of the receptacle, DNA content, chromosome differences and nodal anatomy. The results showed that the highest divergence is observed in *trnH-psbA* (20.8 ± 12.2), followed by ITS2 (5.7 ± 3.2). The phylogenetic tree construction using Bayesian analysis showed a divergent boundary between the two species suggesting that *F.krishnae* could be an independent species, not a variety of *F.benghalensis*. The present study’s findings support the view that these two floras can be treated as different species.

## ﻿Introduction

*Ficus* L. (Fig) is one of the largest and most diverse genera of angiosperms in the family Moraceae, comprising 750 species, mainly distributed in tropical and subtropical regions of the world (Berg 1989; Berg and Corner 2005; Pederneiras et al. 2015). Fig. trees were characterized by a unique kind of closed inflorescence named syconia and provide a breeding place for fig-wasp pollinators (Wiebes 1979). Recently, the checklist of Indian *Ficus* species was updated, and reported about 89 species and 26 infraspecific taxa, mainly distributed in north Eastern states, peninsular regions, and Andaman and Nicobar Islands (Chaudhary et al. 2012). The major flaw in the systematics of this genus is the high degree of intra-specific variability, which often leads to misidentification and closely related species treated as different taxa (Weiblen 2000; Chantarasuwan et al. 2015). Those species showing morphological similarity are considered as Taxonomically Complex Group Species (TCGs). The morphological characteristics of *Ficus* are not natural and revealed several parallel transitions in growth habits and breeding systems (Machado et al. 1996; Weiblen 2000; Jousselin et al. 2003; Rønsted et al. 2005; Rønsted et al. 2008). The species with uniparental reproduction (e.g. self-fertilization, apomixes, gynogenesis and hybridogenesis) also leads to species complexity and misidentification. Differences in the breeding system among the species of *Ficus*, which is the leading cause for the complexity related to ascertaining the taxonomic status of *Ficus* species, remain poorly understood (Chantarasuwan et al. 2013; Lu et al. 2016).

*Ficuskrishnae* C. De Candolle (1906: t. 8092) belongs to subsection Conosycea under the section Cordifoliae G.Don of the subgenus Spherosuke Raf. (Chaudhary et al. 2012; Pederneiras et al. 2015). This species was highly sacred due to the cup-shaped leaf bases, which were considered “Krishna as the Divine Child, sucking his toe and lying on a banyan leaf.” The species precisely differentiated from similar species *F.benghalensis*Linnaeus (1753: 1059) predominantly based on leaf peculiarities and in addition to the differences in tomentum, aerial roots, stipules, petiole, ostiolar bracts, stamens, stigma and pollinator wasps (Prain 1906; Weiblen 2000). Moreover, a detailed taxonomic description of *F.krishnae* was published in ‘Curtis Botanical Magazine’ (Candolle 1906). Molisch (1930) and Biswas (1932, 1935) described that the peculiar nature of its leaves is due to the bud mutation in *F.benghalensis*. Berg and Corner (2005) had subsumed *F.krishnae* with *F.benghalensis* based on morphological characteristics without considering the leaf characters. Corner (1960a, 1960b, 1961, 1962, 1965) and Chaudhary et al. (2012) have treated *F.krishnae* as a taxonomic variety of *F.benghalensis*. In an earlier study, Priyadarsanan (1999) suggests that the two species are pollinated by different species of wasps, indicating the taxonomic dissimilarity between them based on the pollination system. In 2009, Rout and Aparajita analyzed ISSR markers of 23 *Ficus* species and reported that the two species are formed in the same cluster (Rout and Aparajita 2009). Recent studies of *F.krishnae* species based on morphology and anatomy revealed the characteristics such as habit, plant height, presence of aerial roots, leaves structure, stipules, lamina, receptacle, male flower and stigma of female flowers are distinct (Tiwari et al. 2015). Anand et al. (2016) reported non-divergence in nuclear ITS sequence between the species, while chloroplast DNA (*rps16* and *atpB*) shows slow divergence among both species.

The emergence of a fast, accurate and efficient technique named “DNA barcoding” has proven reliable for identifying both intraspecific and interspecific species and for population studies (Hebert et al. 2003). In plants, several coding and noncoding regions of chloroplast DNA (*rbcL*, *matK, ycf1, ycf5, trnL, rpoc1, trnH-psbA*) (Dong et al. 2015) and internal transcribed spacer (ITS) region of nuclear ribosomal DNA have been recommended as a potential DNA barcode candidate (Baldwin 1992). In 2009, CBOL-Plant Working Group suggests *rbcL* and ITS are the core barcode candidates for all plants species and *trnH-psbA* have been recommended as supplementary barcode candidate. In the case of *Ficus* species, only a few reports assert the molecular studies and suggests nuclear internal transcribed spacer (ITS2) and plastid intergeneric spacer (*trnH-psbA)* are the efficient barcode candidates for differentiating *Ficus* species as it had more variable regions. Besides, intergeneric spacer *trnH-psbA* shows high variable regions, especially in closely allied species (Weiblen 2000; Rønsted et al. 2008; Olivar et al. 2014; [Bibr B9][Bibr B11] and Mahima et al. 2020).

Meanwhile, there is a brewing conflict among taxonomists regarding the taxonomic status of the two species because of the enormous scarcity value attached to the taxa and their high cultural importance, especially in India. Hence we propose that the species complex needs to be evaluated using morphological characteristics and molecular markers to identify the species delimitation and genetic divergence.

## Materials and methods

### Sample collection

**Figure 1.**
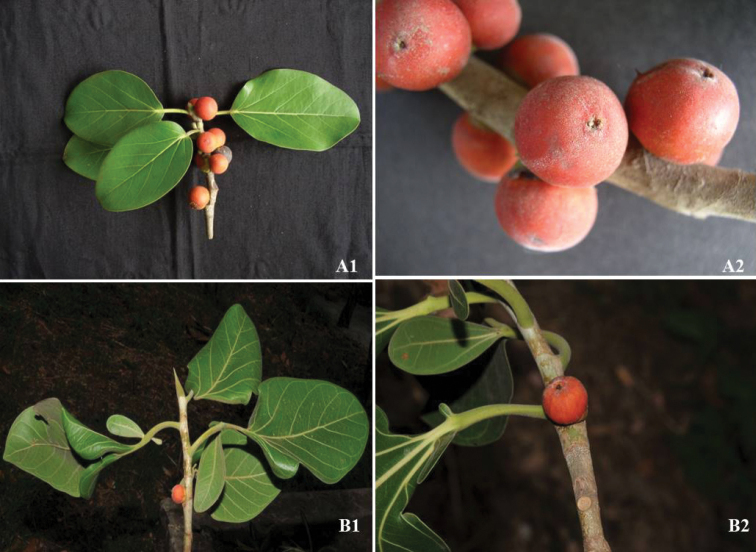
Typical morphology of *Ficusbenghalensis* and *Ficuskrishnae*. **A1**, **A2***Ficusbenghalensis* twig and figs (PC: Jana Venkata Sudhakar) **B1**, **B2***Ficuskrishnae* twig and figs (PC: Jana Venkata Sudhakar)

Fresh leaf samples of both species were collected from A. J. C. Bose Indian Botanic Garden, Howrah, West Bengal; Coimbatore and Calicut University Botanical Garden, Calicut during the period of 2016-17 (Fig. 1). The voucher specimens of *F.benghalensis* and *F.krishnae* (JV135330, JV135335, JV135440, and JV135445) were generated and deposited at the Botanical Survey of India (BSI), Coimbatore, Tamil Nadu, India.

### DNA Extraction, PCR Amplification and DNA Sequencing

About 100 mg of leaf tissue from each sample was used for genomic DNA extraction using Nucleospin Plant II mini spin DNA extraction kit (Macherey-Nagel, Germany), following the manufacturers’ protocol. The purity and quantity of genomic DNA were determined using a spectrophotometer (Nanodrop 2000, Thermo Scientific, USA). The Polymerase Chain Reaction (PCR) was performed for ITS2 (nrDNA) and *trnH-psbA* (cpDNA) in a thermal cycler (Bio-Rad, USA) with 1 µL (50 ng) of DNA product, 10 µL of EmeraldAmp GT PCR master mix (TaKaRa, Bio USA, Inc.), 2 µL each of 10 µM primers (forward and reverse), and 7 µL of nuclease-free H_2_O, in a total volume of 20 µL. The employed primer sets and PCR programs are summarized in (Suppl. material 1: Table S1). The amplified PCR products were inspected on 1% TAE agarose gels and were purified and sequenced on 3730 XL-automated DNA Sequencer by Bioserve Biotechnologies Pvt. Ltd., Hyderabad, Telangana, India. Sequencing was done with both the primers to retrieve the entire length of the desired region.

### DNA sequence alignments

Sequences were initially edited and assembled using Codon Code Aligner version 7.1 (Codon Code Corporation, USA). This software automatically removes the low-quality sequence at the start and end of the sequence using sequence quality scores to identify the low-quality regions. The Phred score of 20 or above was set for the quality check of the sequences. Both forward and reverse primers were trimmed from the assembled sequences and the edited sequences were then aligned using the MUSCLE algorithm in the MEGA 7.0 package (Kumar et al. 2016). BLAST analyses were performed for all the assembled sequences obtained from GenBank to check the potential contamination with non-*Ficus* species DNA and the threshold value of 99% identity was set for the top match (Altschul et al. 1990). The gaps were treated as missing data and indels were excluded from the alignments since they were not informative. Both the strands were sequenced for multiple accessions per species and other *Ficus* species sequences were deposited in GenBank, USA, with the accession number listed in (Suppl. material 2: Table S2). The additional ITS2 and *trnH-psbA* sequences for the following species are *F.racemosa*, *F. religiosa, F. drupacea* and *F.elastica*, and were retrieved from the GenBank. Information pertaining to the sequences data of the present study is provided in Suppl. material 3: Table S3 (Roy et al. 2010; Li et al. 2012; Ando et al. 2016; Williams et al. 2017; Zhang et al. 2018). *F.racemosa* was used as outgroup (Roy et al. 2010).

### Morphological data

In total, 48 qualitative and quantitative characters were selected and coded for the analysis (Suppl. material 4: Table S4). The distinctive characters of *F.krishnae* and *F.benghalensis* are tabularized in (Table 1). The data matrix with multi-state characters are summarized in (Suppl. material 5: Table S5). All characters were treated as unordered and of equal weight, missing data were coded as unknown. The morphological data matrices were constructed using the recent taxonomic revision of *F.krishnae* and *F.benghalensis* (Tiwari et al. 2015). The specimens used in the revision were also the primary source for the compiling data matrix.

**Table 1.** Morphological characteristics feature in *Ficuskrishnae* and *Ficusbenghalensis* species

**S.No**	**Characters**	***F.krishnae* C.DC.**	***F.benghalensis* L.**
1	Habitat	Only in cultivation	Wild as well as in cultivation
2	Plant height	10-15 m tall	25-30 m tall
3	Aerial roots	Few, delicate, thin, do not touch the ground and not forming any accessory trunks	Numerous, strong, thick touch the ground and forming numerous accessories pillar like trunks
4	Stipule	2-6.8 cm long	2.5-3 cm long
5	Petiole	4.5-10 cm long, terete in upper portion and subflat and pulvinous towards base	2-4 cm long, grooved above throughout
6	Leafy appendages on petiole	Generally present	Always present
7	Lamina	Generally, with cup shaped structure on lower surface at base, cuneate at base, entire or sub undulate along margins, downy	Never with cup like structures, acute, obtuse or sub cordate at base, entire along margins, puberulous
8	Receptacle (fig body)	Slightly projected at apex	Dispersed at apex
9	Male flower	Chiefly confined to ostiolar region	Scattered throughout
10	Stigma of female flowers	Linear, feathery or flattened	Linear and swollen, never feathery
11	Chromosome numbers	2n =26 with 1-2 small euchromatic accessory chromosomes	2n=26
12	DNA content	1.47 pg	1.45 pg
13	Nodal anatomy	Multilacunar (7-8 nodal lacunae and their respective traces) and variable	Pentacular (5-nodal lacunae and their respective traces) and constant
14	Size of stomata	Smaller	Larger
15	Size of parenchyma cells	Smaller	Larger

### Phylogenetic analysis

In total, five analyses were done for eight species (three accessions per species), including morphological characteristics evaluation, individual marker ITS2 and *trnH-psbA*, combined marker (ITS2+*trnH-psbA*) were performed with Bayesian Inference (BI) methods. Further, morphological datasets combined with molecular data (morphology+combined markers) as the ‘total evidence’ approach. The morphological dataset was analyzed under the Maximum Parsimony (MP) method using PAUP v4.0b10 (Swofford 2002). Molecular-based analyses were performed in Bayesian Inference (BI) method using MrBAYES v 3.1.2 (Ronquist et al. 2012). For the phylogenetic tree construction, two individual alignment matrices (ITS2 and *trnH-psbA*) and the combined matrices (ITS2+*trnH-psbA*) were aligned separately using the MUSCLE algorithm in the MEGA 7.0 package (Kumar et al. 2016) and checked manually. Model selection for Bayesian analysis was conducted using j Model Test 2.1.7 (Darriba et al. 2012). The models were selected based on BIC scores (Bayesian Information Criterion) and it resulted as HKY for ITS2, TPM3uf+G for *trnH-psbA* and F81+G for combined ITS2+*trnH-psbA*. MP analyses for morphological characters were run in PAUP 4.0b10. In Bayesian phylogenetic analysis, the default values of four chains (three heated and one cold chain) and two independent runs of Markov Chain Monte Carlo (MCMC) for ten million generations with sampling every 1000 generations were performed on the concatenated data matrix. The chain convergence was performed based on the average standard deviation of split frequencies and estimated sample size (ESS) values. A majority-rule consensus tree was then calculated after discarding the first 25% trees as burn-in. The Potential Scale Reduction Factors (PSRF) in the MrBayes SUMP output was one or close to one, describing precise convergence. Bayesian inference produces a moderately higher posterior probability than the equivalent bootstrap frequencies (Erixon et al. 2003); thus, we used only posterior probabilities (PP) above 0.9 as high support. The posterior probabilities values (PP) between 50 and 70 were considered weak support, with percentages between 71 and 89 as moderate, while more than 90 as high support. Fig. tree v.1.4.3 annotator was used to visualize and annotate the Maximum Clade Credibility (MCC) tree from the run subsequently (Rambaut 2018). In addition, interspecies and intra-species divergences were estimated based on the Kimura 2-parameter distance method in the MEGA 7.0 package (Kumar et al. 2016).

## Results

### Species divergence

The amplification and sequence success rate of *F.krishnae* and *F.benghalensis* for the ITS2 and *trnH-psbA* was 100%. The length of the ITS2 and *trnH-psbA* sequences had an average of 328 bp and 317 bp, respectively. The variable informative sites and parsimony-informative sites were high in *trnH-psbA* (13.5%, 15.5%) as compared to ITS2 (7.4%, 8.4%). In order to estimate the genetic divergences between the species, three matrices (mean inter-specific distances, mean intra-specific distances and theta prime) were used; the worked-out results showed that *trnH-psbA* exhibited significant divergences, which aided to distinguish the two species. The mononucleotides ‘A’ or ‘T’ in the *trnH-psbA* sequence did not affect the sequencing and sequence length, respectively. In addition, the calculation of genetic divergence for individual markers in both species showed the absence of any intra-specific divergence in *F.benghalensis* and *F.krishnae*. However, the highest divergence in total inter-specific divergence was observed in *trnH-psbA* (20.8 ± 12.2), followed by ITS2 (5.7 ± 3.2) (Table 2). The multiple sequence alignments of these samples revealed the sequence divergence of *trnH-psbA* to be higher in both species than ITS2, as shown in Suppl. material 6: Fig. S1 and Suppl. material 7: Fig. S2.

**Table 2.** Properties of the two candidate DNA barcoding loci in *Ficuskrishnae* and *Ficusbenghalensis*

**Parameter**	***ITS2***	***trnH-psbA***
PCR Success (%)	100	100
Sequencing success (%)	100	100
Conserved Sites (%)	91	84.5
Variable informative sites (%)	7.4	13.5
Parsimony-informative sites (%)	8.4	15.5
Aligned length (bp)	309	382
No: of Indels	1	26
Identical sites (%)	76.6	29.5
Transition and Transversion bias (R)	0.446	0.51
Mean inter-specific distance (%)	5.7 ± 3.2	20.8 ± 12.2
Mean intra-specific distance (%)	0.2 ± 0.1	0.3 ± 0.1
Theta prime	0.161 ± 0.069	0.494 ± 0.321
BIC model	HKY	TPM3uf+G
Resolution of species (%)	100	100

### Analysis of morphological data

The morphological differences between *F.krishnae* and *F.benghalensis* are tabularized in Table 2. A total of 48 morphological characters, including quantitative and qualitative characters, were coded for analysis. The Maximum Parsimony shown in 1105 trees with a length = 276, consistency index (CI) = 0.27 and retention index (RI) = 0.73. The resulting strict consensus tree presented that two species are partially distinct in morphological characters (Suppl. material 8: Fig. S3).

### Phylogenetic analysis

**Figure 2.**
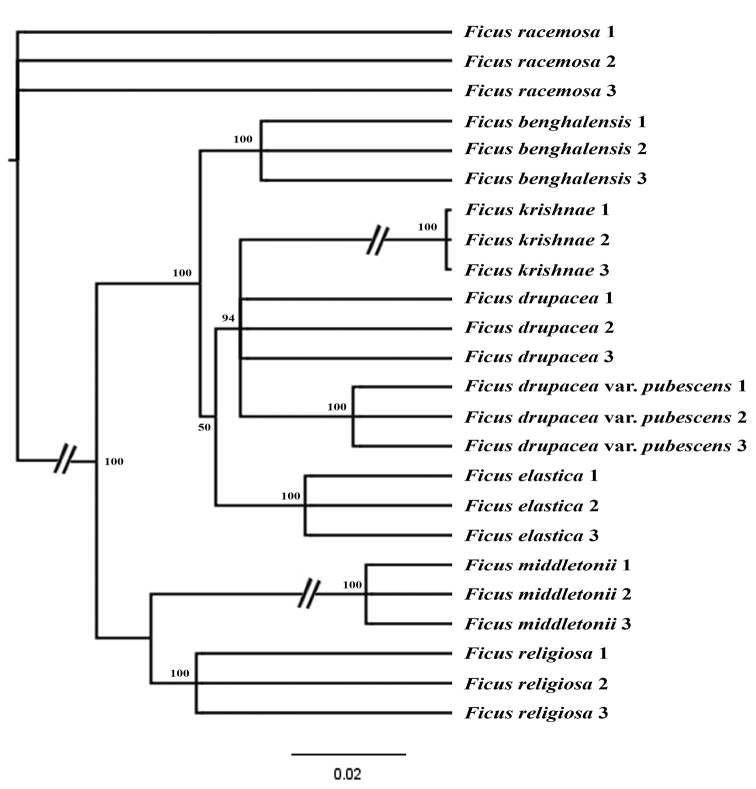
Maximum Clade Credibility (MCC) tree from Bayesian analysis using two DNA barcode markers (ITS2+*trnH-psbA*) with posterior probabilities values in percentage that are shown at nodes.

**Figure 3.**
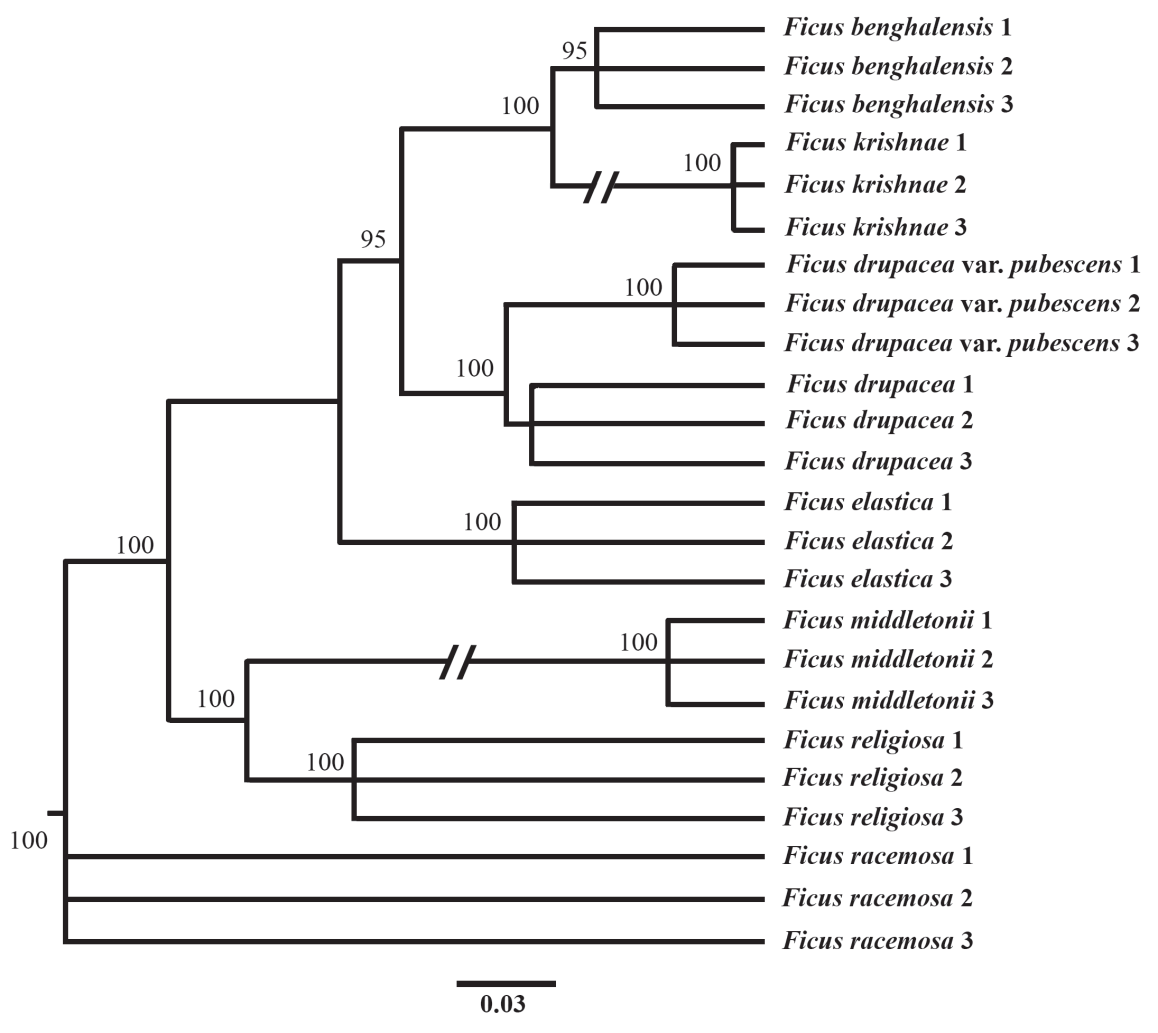
Total evidence MCC tree from Bayesian analysis of the two DNA markers and morphology. The Posterior Probabilities values in percentage are shown at the nodes.

In the present study, a total of 24 taxa (3 accessions per species) with 697 characters, 649 combined ITS2 and *trnH*-psbA sequences data and 48 morphological characters were used for phylogenetic tree construction. The tree of individual marker ITS2 and *trnH-psbA* was generated with low discrimination species resolution (Suppl. material 9: Fig. S4; Suppl. material 10: Fig. S5). The trees of a combined dataset of ITS2+*trnH-psbA* were discriminated with high bootstrap supports (Fig. 2). Considering the conflict between the species, the two markers and morphological characters were combined to analyse Bayesian Inference (BI). The cladogram shows that *F.krishnae* was clearly differentiated from *F.benghalensis*, which formed a separate clade. The branch support with high PP values (100%) indicates that *F.benghalensis* and *F.krishnae* are two distinct lineages and the individuals of the same were bifurcated into two different clades (Fig. 3). Accessions of *F. religiosa, F. middletonii, F. drupacea, F. drupacea* var. *pubescens, F.elastica* and *F.racemosa* were with high posterior probabilities (PP=100), where *F.racemosa* was used as outgroup in Bayesian analysis.

## Discussion

We observed that *F.krishnae* and *F.benghalensis* are morphologically distinct in their characters, such as aerial roots, stipules, petioles, lamina shape, male flower, the stigma of the female flower, and receptacle, but information on their genetic variations is lacking. Recently, DNA barcoding has been implied to identify intra-specific delimitations and resolve taxonomic-complexity based on genetic divergence (Kress et al. 2005; Ragupathy et al. 2009; Nithaniyal and Parani 2016; Sivaraj et al. 2018). Thus, we have also employed a phylogenetic tool to resolve species complexity in *F.benghalensis* with its counterpart *F.krishnae*, based on spacer markers. The morphological characters of *F.krishnae*, such as the height of plants, aerial roots, stipules, petioles and ostiolar bracts of the receptacle, show differentiation from *F.benghalensis* (Tiwari et al. 2015). Other reports supported to the species differentiation in *F.krishnae* as a distinct species from *F.benghalensis*–based on the karyotype (Joshi and Raghuvanshi 1970), DNA contents (Ohri and Khoshoo 1987), stomatal, parenchymatous cells and nodal anatomy (Chattopadhyay and Maiti 2006). Tiwari et al. 2015 considered *F.krishnae* as a distinct species against the merger with *F.benghalensis* under its variety (Berg and Corner, 2005).

Regarding the conflict between species’ distinctness, our results of individual DNA markers (ITS2 and *trnH-psbA*) have shown that the two species are entirely distinct with significant sequence variations (Suppl. material 6: Fig. S1 and Suppl. material 7: Fig. S2). The combined marker studies also revealed the species differences in both species (Fig. 2). Further, we combined the morphology and molecular data to construct a phylogenetic tree, which strongly supports that the two species are distinct (Fig. 3). Thus, morphology and molecular data strongly support each other and derived the concordance. On the other hand, Biswas (1935) reported that the variations in *F.krishnae* were due to bud mutations. Some reports in *Ficus* species show that the evaluation of morphological characteristics combined with molecular analysis is necessary for exact *Ficus* species identification and classification (Chantarasuwan et al. 2015). Lu et al. 2016 reported the taxonomic delimitation of the Hairy-Fig. complex species using phylogenetic analysis and results showed that *F.hirta*, *F.esquiroliana*, *F.simplicissima* and *F.fulva*, with continuously variable morphological characteristics and conspecific based on their genetic traits. In addition, Zhang et al. (2018) studied the *F.auriculata* complex, which contained five species, and resolved the species boundaries using molecular markers and SSR analysis. So hence, we propose that *F.krishnae* is a distinct species among *F.benghalensis* complex due to the possession of high divergence and lineated as sister to the same; it was not nested within *F.benghalensis*. The synonymy of the *F.krishnae* under the *F.benghalensis* complex can be illegitimate for their nomenclature.

## Conclusion

The present study showed that ITS2 and *trnH-psbA* DNA barcode markers could be used as taxon-specific markers for *Ficus* to confirm the species identity. In addition, DNA barcodes will be much more helpful and have been proved to be an effective taxonomic tool for reliable identification, discrimination and resolution among the two closely related *Ficus* L. species. So we suggest that the combination of these two markers with morphology can be used in species delimitation studies to resolve the complexity.

## Supplementary materials

Supplementary material 1 Table S1. The PCR reaction conditions for the barcoding loci Data type molecular data https://binary.pensoft.net/file/620922This dataset is made available under the Open Database License (http://opendatacommons.org/licenses/odbl/1.0/). The Open Database License (ODbL) is a license agreement intended to allow users to freely share, modify, and use this Dataset while maintaining this same freedom for others, provided that the original source and author(s) are credited.Karthikeyan Mahima, Senthilkumar Umapathy, Jana Venkata Sudhakar, Ramalingam Sathishkumar

Supplementary material 2 Table S2. List of *Ficus* species collected from different parts of India Data type species data https://binary.pensoft.net/file/620923This dataset is made available under the Open Database License (http://opendatacommons.org/licenses/odbl/1.0/). The Open Database License (ODbL) is a license agreement intended to allow users to freely share, modify, and use this Dataset while maintaining this same freedom for others, provided that the original source and author(s) are credited.Karthikeyan Mahima, Senthilkumar Umapathy, Jana Venkata Sudhakar, Ramalingam Sathishkumar

Supplementary material 3 Table S3. List of *Ficus* sequences retrieved from GenBank Data type molecular data https://binary.pensoft.net/file/620924This dataset is made available under the Open Database License (http://opendatacommons.org/licenses/odbl/1.0/). The Open Database License (ODbL) is a license agreement intended to allow users to freely share, modify, and use this Dataset while maintaining this same freedom for others, provided that the original source and author(s) are credited.Karthikeyan Mahima, Senthilkumar Umapathy, Jana Venkata Sudhakar, Ramalingam Sathishkumar

Supplementary material 4 Table S4. Vegetative and floral characters Data type species data https://binary.pensoft.net/file/620925This dataset is made available under the Open Database License (http://opendatacommons.org/licenses/odbl/1.0/). The Open Database License (ODbL) is a license agreement intended to allow users to freely share, modify, and use this Dataset while maintaining this same freedom for others, provided that the original source and author(s) are credited.Karthikeyan Mahima, Senthilkumar Umapathy, Jana Venkata Sudhakar, Ramalingam Sathishkumar

Supplementary material 5 Table S5. The data matrix of vegetative and floral characters Data type species data https://binary.pensoft.net/file/620926This dataset is made available under the Open Database License (http://opendatacommons.org/licenses/odbl/1.0/). The Open Database License (ODbL) is a license agreement intended to allow users to freely share, modify, and use this Dataset while maintaining this same freedom for others, provided that the original source and author(s) are credited.Karthikeyan Mahima, Senthilkumar Umapathy, Jana Venkata Sudhakar, Ramalingam Sathishkumar

Supplementary material 6 Figure S1. Multiple sequence alignment of *ITS2* in *Ficuskrishnae* and *Ficusbenghalensis* Data type molecular data https://binary.pensoft.net/file/620927This dataset is made available under the Open Database License (http://opendatacommons.org/licenses/odbl/1.0/). The Open Database License (ODbL) is a license agreement intended to allow users to freely share, modify, and use this Dataset while maintaining this same freedom for others, provided that the original source and author(s) are credited.Karthikeyan Mahima, Senthilkumar Umapathy, Jana Venkata Sudhakar, Ramalingam Sathishkumar

Supplementary material 7 Figure S2. Multiple sequence alignment of *trnH-psbA* in *Ficuskrishnae* and *Ficusbenghalensis* Data type molecular data https://binary.pensoft.net/file/620928This dataset is made available under the Open Database License (http://opendatacommons.org/licenses/odbl/1.0/). The Open Database License (ODbL) is a license agreement intended to allow users to freely share, modify, and use this Dataset while maintaining this same freedom for others, provided that the original source and author(s) are credited.Karthikeyan Mahima, Senthilkumar Umapathy, Jana Venkata Sudhakar, Ramalingam Sathishkumar

Supplementary material 8 Figure S3. Parsimony distribution of the morphological characters and their status on the MCC Bayesian tree Data type molecular data https://binary.pensoft.net/file/620930This dataset is made available under the Open Database License (http://opendatacommons.org/licenses/odbl/1.0/). The Open Database License (ODbL) is a license agreement intended to allow users to freely share, modify, and use this Dataset while maintaining this same freedom for others, provided that the original source and author(s) are credited.Karthikeyan Mahima, Senthilkumar Umapathy, Jana Venkata Sudhakar, Ramalingam Sathishkumar

Supplementary material 9 Figure S4. Maximum Clade Credibility (MCC) tree from Bayesian analysis using the ITS2 DNA barcode marker with posterior probabilities values in percentage that are shown at nodes Data type molecular data https://binary.pensoft.net/file/620931This dataset is made available under the Open Database License (http://opendatacommons.org/licenses/odbl/1.0/). The Open Database License (ODbL) is a license agreement intended to allow users to freely share, modify, and use this Dataset while maintaining this same freedom for others, provided that the original source and author(s) are credited.Karthikeyan Mahima, Senthilkumar Umapathy, Jana Venkata Sudhakar, Ramalingam Sathishkumar

Supplementary material 10 Figure S5. Maximum Clade Credibility (MCC) tree from Bayesian analysis using the *trnH-psbA* DNA barcode marker with posterior probabilities values in percentage that are shown at nodes. Data type NEX file. https://binary.pensoft.net/file/620932This dataset is made available under the Open Database License (http://opendatacommons.org/licenses/odbl/1.0/). The Open Database License (ODbL) is a license agreement intended to allow users to freely share, modify, and use this Dataset while maintaining this same freedom for others, provided that the original source and author(s) are credited.Karthikeyan Mahima, Senthilkumar Umapathy, Jana Venkata Sudhakar, Ramalingam Sathishkumar
