# Supplementary material for: Systematic reinstatement of highly sacred Ficuskrishnae based on differences in morphology and DNA barcoding from Ficusbenghalensis (Moraceae)
Source: PhytoKeys. 2021 Dec 9;186:121–38. doi: 10.3897/phytokeys.186.74086 (PMC8677708; doi:10.3897/phytokeys.186.74086)
Supplement: Supplementary material 1 — Table S1. The PCR reaction conditions for the barcoding loci [file phytokeys-186-121-s001.pdf]

Table S1. The PCR reaction conditions for the barcoding loci.

**Loci**	**Initial denaturation**	**Denaturation**	**Annealing**	**Extension**	**Final Extension**	**Total no: of cycles**
ITS2	94°C–5 min	94°C– 1min	58°C–1 min	72°C–1:30 min	72 °C–10 min	30
trnH - *psbA*	94 °C–5 min	94 °C–1 min	55 °C–1 min	72 °C –1: 30 min	72 °C –10 min	25
