# Supplementary material for: Systematic reinstatement of highly sacred Ficuskrishnae based on differences in morphology and DNA barcoding from Ficusbenghalensis (Moraceae)
Source: PhytoKeys. 2021 Dec 9;186:121–38. doi: 10.3897/phytokeys.186.74086 (PMC8677708; doi:10.3897/phytokeys.186.74086)
Supplement: Supplementary material 2 — Table S2. List of Ficus species collected from different parts of India [file phytokeys-186-121-s002.pdf]

Table S2. List of *Ficus* species collected from different parts of India.

Voucher ID	Name of the species	District	State	GenBank Accession No
JV135330	*Ficus benghalensis*	A. J. C. Bose Indian Botanic Garden, Howrah	West Bengal (WB1)	KU365060
JV135335	*Ficus benghalensis*	Bharathiar University, Coimbatore	Tamil Nadu (TN1)	KX173433
JV135440	*Ficus .krishnae*	A. J. C. Bose Indian Botanic Garden, Howrah	West Bengal (WB1)	KU365061
JV135445	*Ficus krishnae*	Calicut University Botanical Garden	Kerala (KL1)	KX173434
JV135330	*Ficus benghalensis*	A. J. C. Bose Indian Botanic Garden, Howrah	West Bengal (WB2)	MF288774
JV135335	*Ficus benghalensis*	Coimbatore	Tamil Nadu (TN2)	MF288780
JV135440	*Ficus krishnae*	A. J. C. Bose Indian Botanic Garden, Howrah	West Bengal (WB2)	MF288775
JV135445	*Ficus krishnae*	Calicut University, Kerala	Kerala (KL2)	MF288781
JV135448	*Ficus middletonii*	Anamalais, Tamil Nadu	Tamil Nadu (TN1)	KU365057
JV135450	*Ficus middletonii*	Nilgiris, Tamil Nadu	Tamil Nadu (TN2)	MN922249

JV135448	*Ficus middletonii*	Anamalai, Tamil Nadu	Tamil Nadu (TN1)	KX173430
JV135448	*Ficus middletonii*	Anamalai, Tamil Nadu	Tamil Nadu (TN1)	MT118725
JV135450	*Ficus middletonii*	Nilgiris, Tamil Nadu	Tamil Nadu (TN2)	MT118726
JV135460	*Ficus drupacea*	Calicut University, Kerala	Kerala (KL2)	MN922243
JV135460	*Ficus drupacea*	Calicut University, Kerala	Kerala (KL2)	MN922244
JV135482	*Ficus racemosa*	Coimbatore, Tamil Nadu	Tamil Nadu (TN1)	MN922253
JV135450	*Ficus middletonii*	Nilgiris, Tamil Nadu	Tamil Nadu (TN2)	Submission in process
JV135495	*Ficus drupacea* var. *pubescens*	Calicut University, Kerala	Kerala (KL2)	Submission in process
JV135495	*Ficus drupacea* var. *pubescens*	Coimbatore, Tamil Nadu	Tamil Nadu (TN2)	Submission in process
JV135496	*Ficus drupacea* var. *pubescens*	Calicut Botanical Garden, Kerala	Kerala (KL1)	Submission in process
JV135496	*Ficus drupacea* var. *pubescens*	Calicut Botanical Garden, Kerala	Kerala (KL1)	Submission in process
JV135495	*Ficus drupacea* var. *pubescens*	Coimbatore, Tamil Nadu	Tamil Nadu (TN2)	Submission in process

JV135495	*Ficus drupacea* var. *pubescens*	Coimbatore, Tamil Nadu	Tamil Nadu (TN2)	Submission in process
JV135445	*Ficus krishnae*	Calicut University Botanical Garden	Kerala (KL1)	Submission in process
JV135440	*Ficus krishnae*	A. J. C. Bose Indian Botanic Garden, Howrah	West Bengal (WB2)	Submission in process
