# Supplementary material for: Systematic reinstatement of highly sacred Ficuskrishnae based on differences in morphology and DNA barcoding from Ficusbenghalensis (Moraceae)
Source: PhytoKeys. 2021 Dec 9;186:121–38. doi: 10.3897/phytokeys.186.74086 (PMC8677708; doi:10.3897/phytokeys.186.74086)
Supplement: Supplementary material 3 — Table S3. List of Ficus sequences retrieved from GenBank [file phytokeys-186-121-s003.pdf]

Table S3: List of *Ficus* species sequences retrieved from GenBank.

Scientific name	GenBank Accession No	Reference
Ficus drupacea	KU855508	Williams et al. 2017
Ficus drupacea	LC461816	Sukrong (unpublished)
Ficus drupacea	KU855509	Williams et al. 2017
Ficus drupacea	KU85507	Williams et al. 2017
Ficus benghalensis	KY700345	Moorhouse-Gann et al (unpublished)
Ficus benghalensis	AB985788	Ando et al. 2016
Ficus racemosa	HM368195	Roy et al. 2010
Ficus racemosa	HM368196	Roy et al. 2010
Ficus racemosa	MG977838	Zhang et al. 2018 (unpublished)
Ficus racemosa	MG977837	Zhang et al. 2018 (unpublished)
Ficus racemosa	MG977836	Zhang et al. 2018 (unpublished)
Ficus religiosa	JQ773965	Li et al. 2012
Ficus religiosa	JQ773964	Li et al. 2012
Ficus religiosa	JQ773963	Li et al. 2012
Ficus religiosa	JQ774205	Li et al. 2012
Ficus religiosa	JQ774204	Li et al. 2012
Ficus religiosa	JQ774203	Li et al. 2012
Ficus elastica	KT726863	Reddy et al. 2015 (unpublished)
Ficus elastica	AB985787	Ando et al 2016
Ficus elastica	JQ774300	Li et al 2012
Ficus elastica	JQ773867	Li et al 2012
Ficus elastica	JQQ77386	Li et al 2012
Ficus elastica	JQ773865	Li et al 2012
