# Supplementary material for: Systematic reinstatement of highly sacred Ficuskrishnae based on differences in morphology and DNA barcoding from Ficusbenghalensis (Moraceae)
Source: PhytoKeys. 2021 Dec 9;186:121–38. doi: 10.3897/phytokeys.186.74086 (PMC8677708; doi:10.3897/phytokeys.186.74086)
Supplement: Supplementary material 4 — Table S4. Vegetative and floral characters [file phytokeys-186-121-s004.pdf]

Table S4. Vegetative and Floral characters.

1. **Habit**: Trees (0) Standing shrubs (1) Bushy-climbing shrubs (2) Procumbent or root climber (3)
2. **Trees different forms:** Sympodial- Terminalia (0) Pachycladous trees (1) Leptocladous-trees (2)
3. **Habitat:** Mesophytes (0) Semi or hemi epiphytes (1) Epiphytes (2) Rheophytes (3) Lithophytes (4) Lianescent (5)
4. **Aerial roots:** Absent (0) Few (1) Numerous (2)
5. **Sexuality:** Monoecious (0) Dioecious (1)
6. **Different forms of Leaves:** Heterophyllus condition absent (0) Heterophyllus condition present (1)
7. **Leaf Phyllotaxy:** Alternate (0) Opposite (1)
8. **Lamina symmetry**: Symmetric (0) Asymmetric (1)
9. **Petioles**: Articulated (0) Not Articulated (1)
10. **Lamina shape**: obovate to spathulate (0) ovate to cordiform (1) linear to elliptic (2) palmately or sagittate lobed (3) rhomboid to subrohomboid or oblanceolate (4)
11. **Lamina size**: 21 to 30 cm (0), < or=1 (1) 1 to 10 cm (2), 11 to 20 cm (3), >30 (4)
12. **Leaf apex**: Acute (0) Obtuse (1) Acuminate (2) Caudate (3)
13. **Leaf base**: Semi-cordate (0) Not semicordata (1) cupuliform or coneshaped (2)
14. **Leaf nature:** Coriaceous (0) Sub-coriaceous (1)
15. **Leaf texture on abaxial surface**: Glabrous (0) Puberulous to pubescent (1) Flocculent (2) Tomentose (3) Scabrous and hispidulous (4)
16. **Margins of lamina**: Entire (0) Serrate (1) Dentate or waved (2)
17. **Venation**: Reticulate venation (0) Unicostate with parallel lateral nerves (1) Multicostate (2) Reticulate, depressed beneath and forms bullate appearance (3)
18. **Petioles length**: 1 to 10 cm (0), < or=1 (1), 11 to 20 cm (2), 21 to 30 cm (3)
19. **Waxy glands :** Absent (0) Present (1)
20. **Waxy glands position**: On the lower surface of lamina (0), Nodes of leafy twigs (1) Rarely on the upper surface (2) Base of midrib or apex of peduncle (3)Axial of basal lateral nerves (4)
21. **Stipules size**: 1 to 10 cm (0), < or=1 (1) 11 to 20 cm (2), 21 to 30 cm (3)
22. **Stipules shape**: Ovate (0) Lanceolate (1) Ovate to lanceolate (2)
23. **Stipules texture**: Glabrous (0) Pubescent (1)
24. **Annular scars**: Absent (0) Present (1)
25. **Cystoliths:** Amphigenous (0) Hypergenous (1) Hypogenous (2) Absent (3)
26. **Inflorescence type**: Sorosis (0) Syconium (1)
27. **Formation of inflorescence:** 1 to 4 (0) solitary (1) clusters(2)
28. **Position of inflorescence**: Cauliflorus (0), Axiliary or below the leaves (1)
29. **Inflorescence nature**: Sorosis present (0) Sessile (1) Subsessile (2) Pedunculate (3)
30. **Inflorescence shape**: Sorosis cylindrical (0) Syconium globose to subglobose or depressed globose (1) Syconium Ellipsoid (2) Syconium obovoid or obconical (3) Syconium oblong (4) Syconium Pyriform (5)
31. **Inflorescence size (in dia.):** > 50 cm (0) 0.5 cm to 5 cm (1) 6 to 10cm (2)

32. **Inflorescence texture**: Glabrous (0) Puberulous (1) Pubescent (2) Tomentose (3) Verrucose (4) Hispid or Scabrid (5)
33. **Inflorescence color:** Greenish or yellowish to brownish (0) Pale to light green (1) Red or pinkish red (2) Yellow to Orange (3) Pink to purple or Dark purple (4)
34. **Basal bracts:** Absent (0) Present (1)
35. **Basal bracts nature**: Caducous (0) Persistent (1)
36. **Peduncular bracts:** Absent (0) Present (1)
37. **Ostiolar bracts:** Absent (0) Present (1)
38. **Interfloral bracts:** Absent (0) Present (1)
39. **Internal hairs or bristles:** Absent (0) Present (1)
40. **Flowers:** Unisexual (0) Bisexual (1)
41. **Tepals nature in Male flowers:** Free (0) United (1) Partly united (2)
42. **Tepals nature in Female flowers:** Free (0) United (1) Partly united (2)
43. **Gall flowers:** Absent (0) Present (1)
44. **Pseudo-hermaphrodite flowers**: Absent (0) Present (1)
45. **Style of female flowers**: Glabrous (0) Puberulous (1)
46. **Peduncle length**: 1 to 10 cm (0) < or=1cm (1) Peduncle absent (2)
47. **Fruit type:** Sorosis with fleshy perianth seeds (0) Achene (1)
48. **Fruit shape**: Oblong(0) Globose (1) Ovoid (2) Ellipsoid (3) Obovoid (4) Lenticular (5) Trigonous (6)
