# Supplementary material for: Systematic reinstatement of highly sacred Ficuskrishnae based on differences in morphology and DNA barcoding from Ficusbenghalensis (Moraceae)
Source: PhytoKeys. 2021 Dec 9;186:121–38. doi: 10.3897/phytokeys.186.74086 (PMC8677708; doi:10.3897/phytokeys.186.74086)
Supplement: Supplementary material 6 — Figure S1. Multiple sequence alignment of ITS2 in Ficuskrishnae and Ficusbenghalensis [file phytokeys-186-121-s006.pdf]

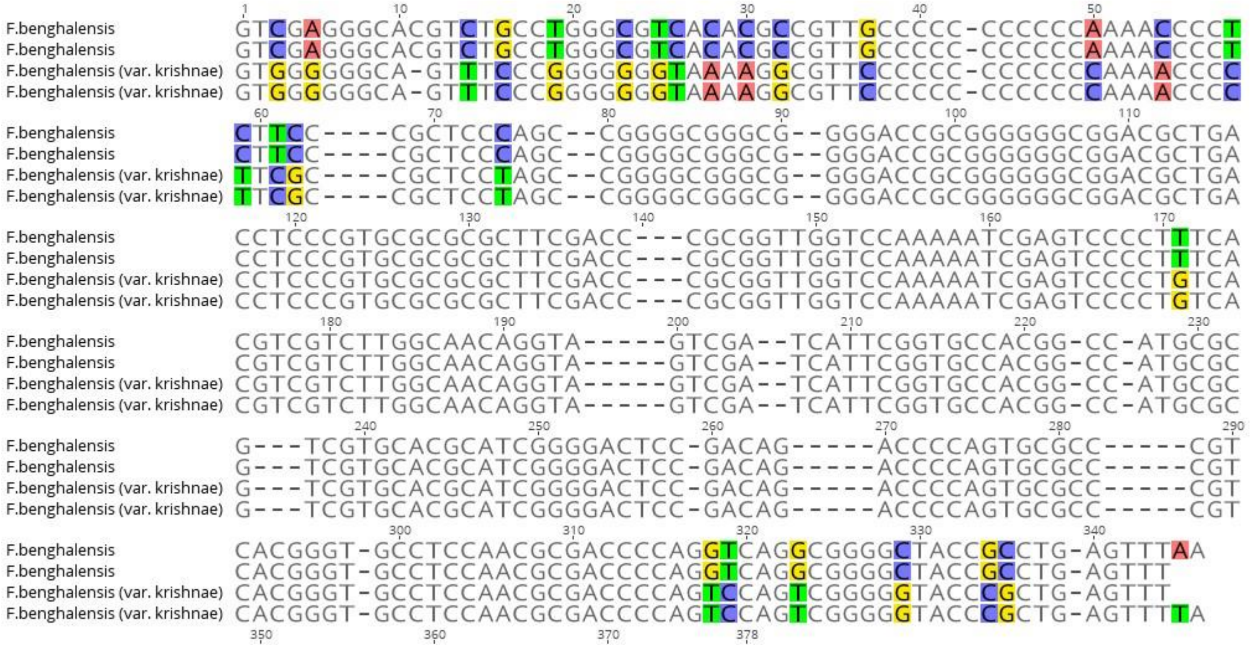

Supplementary Figure 1: Multiple sequence alignment of ITS2 in *Ficus krishnae* and *Ficus benghalensis.*
