# Supplementary material for: Systematic reinstatement of highly sacred Ficuskrishnae based on differences in morphology and DNA barcoding from Ficusbenghalensis (Moraceae)
Source: PhytoKeys. 2021 Dec 9;186:121–38. doi: 10.3897/phytokeys.186.74086 (PMC8677708; doi:10.3897/phytokeys.186.74086)
Supplement: Supplementary material 8 — Figure S3. Parsimony distribution of the morphological characters and their status on the MCC Bayesian tree [file phytokeys-186-121-s008.pdf]

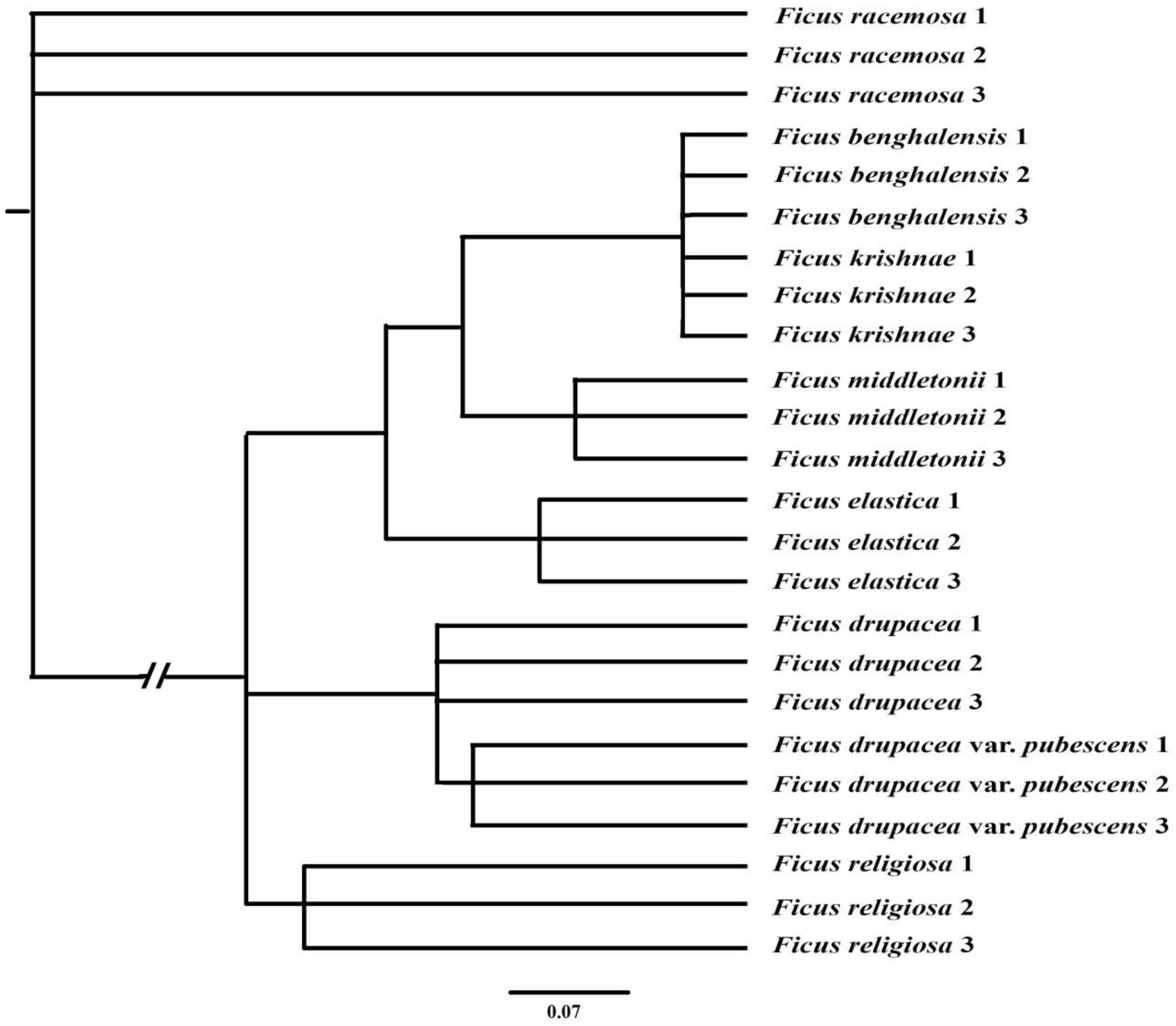

Supplementary Figure 3: Parsimony distribution of the morphological characters and their status on the MCC Bayesian tree.
