# Supplementary material for: Systematic reinstatement of highly sacred Ficuskrishnae based on differences in morphology and DNA barcoding from Ficusbenghalensis (Moraceae)
Source: PhytoKeys. 2021 Dec 9;186:121–38. doi: 10.3897/phytokeys.186.74086 (PMC8677708; doi:10.3897/phytokeys.186.74086)
Supplement: Supplementary material 10 — Figure S5. Maximum Clade Credibility (MCC) tree from Bayesian analysis using the trnH-psbA DNA barcode marker with posterior probabilities values in percentage that are shown at nodes. [file phytokeys-186-121-s010.pdf]

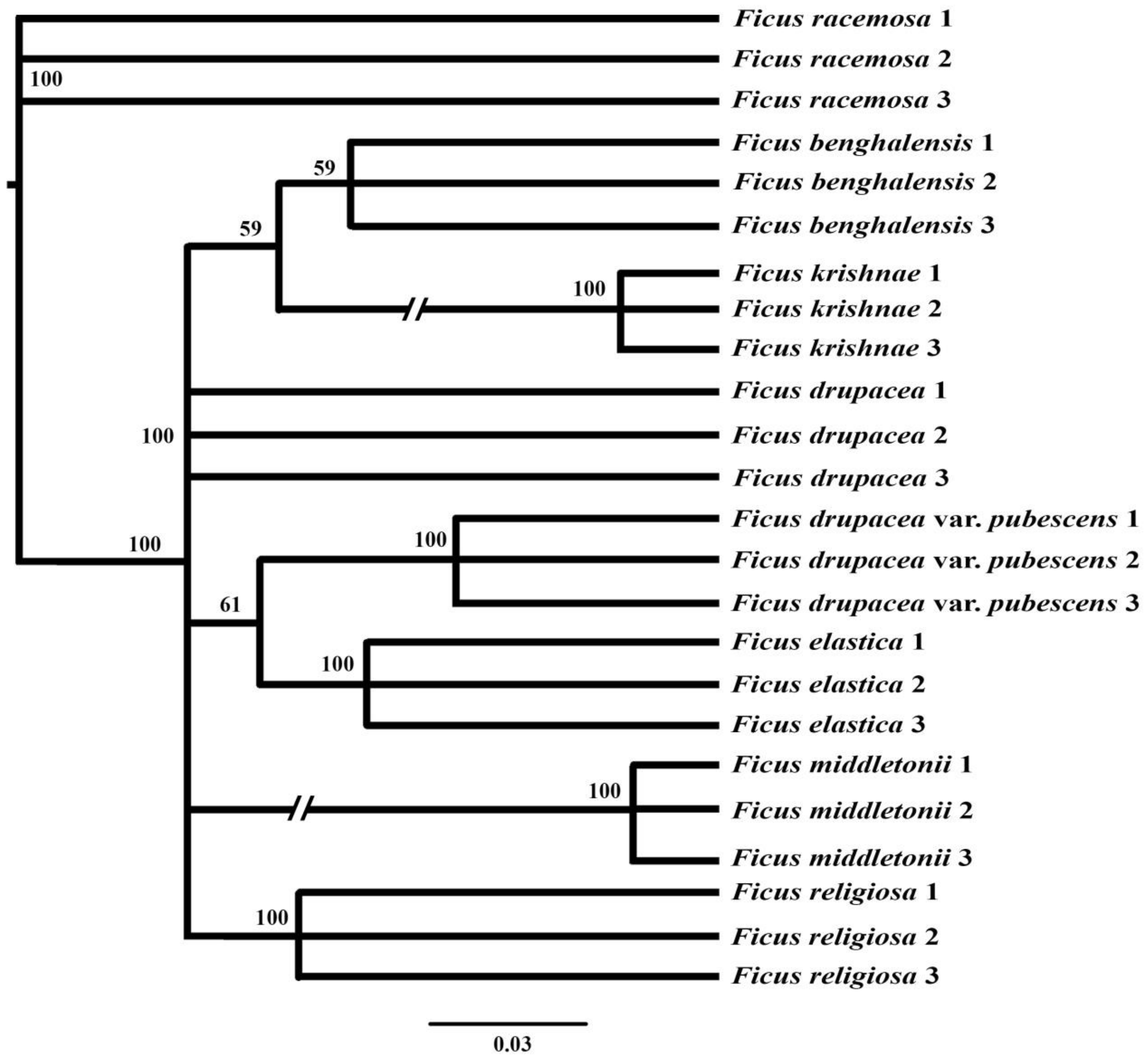

Supplementary Figure 5: Maximum Clade Credibility (MCC) tree from Bayesian analysis using the *trnH-psbA* barcode marker with posterior probabilities values in percentage that are shown at nodes.
